# Atg14: A Key Player in Orchestrating Autophagy

**DOI:** 10.1155/2011/713435

**Published:** 2011-10-13

**Authors:** Keisuke Obara, Yoshinori Ohsumi

**Affiliations:** ^1^Faculty of Pharmaceutical Sciences, Hokkaido University, Kita-12 Jo Nishi-6 Chome, Kitaku, Sapporo 060-0812, Japan; ^2^Frontier Research Center, Tokyo Institute of Technology, 4259-S2-12 Nagatsuda-Cho, Midoriku, Yokohama 226-8503, Japan

## Abstract

Phosphorylation of phosphatidylinositol (PtdIns) by a PtdIns 3-kinase is an essential process in autophagy. Atg14, a specific subunit of one of the PtdIns 3-kinase complexes, targets the complex to the probable site of autophagosome formation, thereby, sorting the complex to function specifically in autophagy. The N-terminal half of Atg14, containing coiled-coil domains, is required to form the PtdIns 3-kinase complex and target it to the proper site. The C-terminal half of yeast Atg14 is suggested to be involved in the formation of a normal-sized autophagosome. The C-terminal half of mammalian Atg14 contains the Barkor/Atg14(L) autophagosome-targeting sequence (BATS) domain that preferentially binds to the highly curved membranes containing PtdIns(3)*P* and is proposed to target the PtdIns 3-kinase complex efficiently to the isolation membrane. Thus, the N- and C-terminal halves of Atg14 are likely to have an essential core function and a regulatory role, respectively.

## 1. PtdIns 3-Kinase in Autophagy

Eukaryotic cells can enclose their own cytoplasmic components in a double-membrane structure, the autophagosome, and deliver it to a lytic compartment, the vacuole/lysosome, where the contents are then degraded. This conserved system is involved not only in the recycling of proteins under starvation conditions but also in the clearance of organelles and aberrant aggregate-prone proteins, digestion of invading pathogens, and so on [1–4]. Genes involved in autophagy were first identified by yeast genetic screenings [5–7]. At present, more than 30 autophagy-related (*ATG*) genes have been identified in yeast, and of them at least 18 genes are essential for autophagosome formation, a crucial process in autophagy. Most of these 18 genes are conserved in mammals, suggesting that the mechanism of autophagosome formation is basically conserved from yeast to mammals. The 18 Atg proteins can be divided into five groups according to their functions [8, 9]. One group consists of subunits of a class III phosphatidylinositol (PtdIns) 3-kinase complex (hereafter, PtdIns 3-kinase indicates the class III PtdIns 3-kinase). Atg14 and Vps30/Atg6 are two such proteins and are included in this group together with Vps34 and Vps15, catalytic and regulatory subunits, respectively, (the functions of Vps34 and Vps15 in the vacuolar protein sorting pathway have been studied in detail, and; thus, they are not designated as Atg proteins although they are essential for autophagy). Atg14 is a key subunit in determining the function of the PtdIns 3-kinase complex and is the focus of this paper (see following sections). 

**Figure 1:**
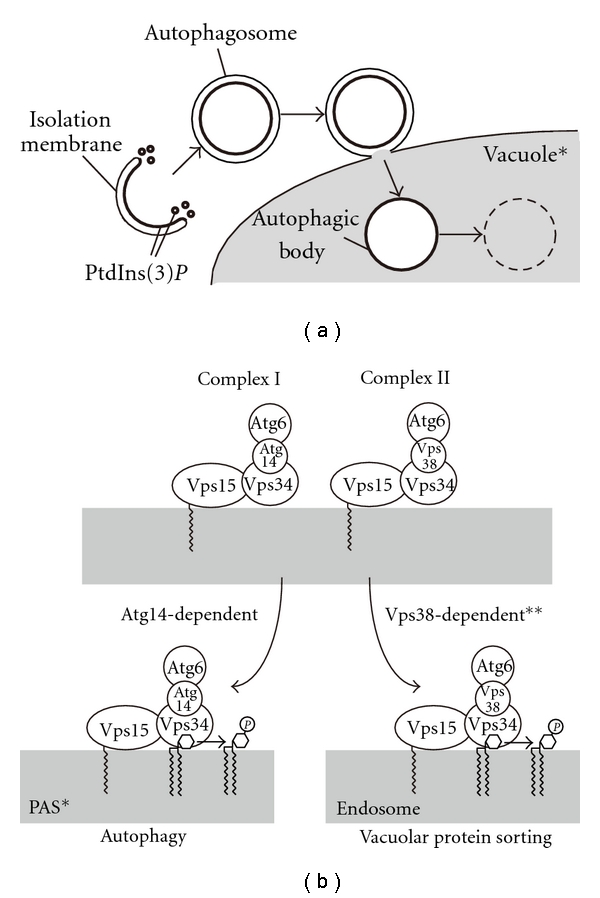
Atg14 is a key factor in determining the function of the PtdIns 3-kinase complex. (a) Membrane dynamics of autophagy and movement of PtdIns(3)*P* in yeast. The isolation membrane extends to enclose the cytoplasmic contents. The closed double-membrane structure, the autophagosome, fuses with the vacuole and releases the inner membrane structure, the autophagic body, into the lumen where the enclosed contents are degraded [25]. PtdIns(3)*P* (bold line) is enriched at the inner surface of the isolation membrane and the autophagosome [26]. PtdIns(3)*P* is also enriched on some uncharacterized amorphous membranes near the tips of the isolation membrane. (*) In mammals the autophagosome fuses with the lysosome, which is often smaller than the autophagosome, and the released lysosomal hydrolytic enzymes degrade the inner membrane structure to become the autolysosome. (b) Assortment of PtdIns 3-kinase complexes in yeast. Yeast has at least two PtdIns 3-kinase complexes (Complexes I and II). Atg14 is a specific subunit of Complex I. Atg14 targets Complex I to the PAS, thereby, providing Complex I with the ability to function in autophagy. In mammals, in addition to Complexes I and II, a third complex exists in which Rubicon is added to Complex II; this complex negatively regulates maturation of the autophagosome. (*) Instead of the PAS, Barkor/Atg14(L) targets the autophagy-specific PtdIns 3-kinase complex to an ER subdomain in mammalian cells. (**) Although endosomal localization of the complete Complex II (including Atg6) is dependent on Vps38, that of Vps34 and Vps15 does not require Vps38. (a) and (b) reproduced from Obara and Ohsumi [26] (Copyright 2008 by Landes Bioscience) and [17] (Copyright 2011 by the Hindawi Publishing Corporation), respectively.

PtdIns 3-kinase phosphorylates PtdIns at the d-3 position of the inositol ring, generating PtdIns(3)*P*. In yeast, Vps34 is the sole PtdIns 3-kinase [10]. Vps34 is essential to both the vacuolar protein-sorting pathway and to autophagy [11, 12]. Autophagic activity is completely abolished in *vps34*Δ cells expressing a lipid kinase-dead form of Vps34, indicating that production of PtdIns(3)*P* is essential for autophagy [13]. In yeast, it was shown that PtdIns(3)*P* is enriched in the inner surface of the isolation membrane and autophagosome (Figure 1) [13]. Produced PtdIns(3)*P* recruits downstream molecules, such as Atg18, that are considered to be directly involved in autophagosome formation [14, 15]. For a general introduction to the function of PtdIns 3-kinase and PtdIns(3)*P* in autophagy, please refer to other reviews [16, 17]. 

PtdIns 3-kinase is essential for autophagy in mammals as well. Inhibitors of PtdIns 3-kinase, such as wortmannin and 3-methyladenine, suppress autophagy in mammalian cells. Knockdown of mammalian *VPS34* also suppresses autophagy [18–21]. Conversely, supplementation with PtdIns(3)*P*, but not other phosphoinositides, enhances autophagic degradation in HT-29 cells [22]. Like in yeast, PtdIns 3-kinase is also required for vesicular trafficking toward the lytic compartment, the lysosome [23, 24].

## 2. Atg14: A Specific Subunit of the PtdIns  3-Kinase Complex Involved in Autophagy

As mentioned above, PtdIns 3-kinase is required for both autophagy and vacuolar protein sorting [11, 12]. The means by which the sole PtdIns 3-kinase, Vps34, is involved in these two distinct processes is explained by the existence of multiple PtdIns 3-kinase complexes (Figure 1(b)). In yeast, Vps34 forms two distinct PtdIns 3-kinase complexes (Complexes I and II) that are involved in different processes [12]. Complex I specifically functions in autophagy while Complex II is required in the vacuolar protein sorting pathway. Both complexes have PtdIns 3-kinase activity and share three common subunits, Vps34, Vps15, and Vps30/Atg6. Vps34 is a catalytic subunit. The PtdIns 3-kinase activity of Vps34 is not required to form the PtdIns 3-kinase complexes but is essential for autophagy and for the vacuolar protein sorting pathway. Vps15 is a serine/threonine protein kinase that phosphorylates Vps34 and recruits it to the membrane fraction [27]. The kinase activity of Vps15 is required to form the PtdIns 3-kinase complexes. Vps15 is myristoylated, but the membrane association of the PtdIns 3-kinase complexes does not solely depend on myristoylation [28]. The precise role of Vps30/Atg6 within the PtdIns 3-kinase complexes is still unclear. However, Beclin 1, a mammalian homolog of Vps30/Atg6, interacts with various proteins involved in other processes and is proposed to serve as a platform upon which multiple cellular signals converge, thereby regulating the balance between autophagy and other biological processes [29, 30]. In addition to the common subunits, each complex contains a unique factor. Atg14 is specifically integrated into Complex I, while Complex II contains Vps38 as a specific subunit. These specific factors play an essential role in sorting the PtdIns 3-kinase complexes to the distinct processes.

## 3. Function of Atg14

Atg14 bridges Vps30/Atg6 and Vps34 to allow formation of Complex I. Similarly, Vps38 bridges Vps30/Atg6 and Vps34 to form Complex II. Thus, these unique subunits serve as connectors to form PtdIns 3-kinase complexes. 

In addition, Atg14 and Vps38 play critical roles in determining the function of PtdIns 3-kinase complexes. Deletion of *ATG14 *does not affect vacuolar protein sorting, and, conversely, disruption of *VPS38* does not suppress autophagy [12, 31]. Overexpression of Vps38 does not restore autophagic activity in *atg14*Δ cells [32]. Thus, the PtdIns 3-kinase complexes are strictly sorted to distinct functions depending on the specific subunits, Atg14 and Vps38.

Atg14 directs Complex I to function in autophagy by regulating its localization [32]. Complex I is present on the vacuolar membrane and at a perivacuolar structure called the preautophagosomal structure (PAS) [33]. Most of Atg proteins localize to the PAS, and; thus, the PAS is considered to be closely related to autophagosome formation. PAS localization of Vps34 (the catalytic subunit) and Vps30/Atg6 is abolished in *atg14*Δ cells, indicating that Complex I is targeted to the PAS in an Atg14-dependent manner (Figure 1(b)). On the other hand, deletion of *VPS38 *does not affect PAS localization of Complex I. Complex II localizes to the vacuolar membrane and the endosome. Whether or not the endosomal localization of Complex II depends on Vps38 is not a simple question. In *vps38*Δ cells, the endosomal localization of Vps30/Atg6 is abolished, but Vps15 and Vps34 still localize to the endosome. Thus, the endosomal localization of the complete Complex II, including Vps30/Atg6, is dependent on Vps38, whereas that of the catalytic and regulatory subunits, Vps34 and Vps15, is not. Localization of Complex II is not affected in *atg14*Δ cells. In summary, Atg14 has at least two functions in autophagy: (i) Atg14 acts as a connector to form the PtdIns 3-kinase complex and (ii) Atg14 directs Complex I to function in autophagy by targeting it to the PAS.

## 4. Discovery of Mammalian Atg14

A simple BLAST database search failed to identify a mammalian homolog of Atg14. Recently; however, several groups succeeded in identifying mammalian Atg14. Itakura et al. [18] identified a candidate for mammalian Atg14 by PSI-BLAST, a method for detecting weak but biologically relevant sequence similarities that is more sensitive than conventional BLAST, and experimentally confirmed that it was the bona fide mammalian Atg14. Other groups identified mammalian Atg14 using a biochemical approach, that is, identifying proteins that are copurified with Beclin 1 (mammalian Atg14 is hereafter called Barkor/Atg14(L)) [19–21]. Similar to yeast Atg14, Barkor/Atg14(L) forms a PtdIns 3-kinase complex with Beclin 1 and mammalian homologs of Vps34 and Vps15. On the other hand, UVRAG, originally identified as a protein related to UV resistance, is considered to be a counterpart for Vps38. Thus, PtdIns 3-kinase complexes that correspond to yeast Complexes I and II exist in mammals. In addition, mammalian cells have another PtdIns 3-kinase complex composed of Vps34, Vps15, Beclin 1, UVRAG, and Rubicon, a Rab7 effecter containing a RUN domain [20, 21]. The Rubicon-containing complex negatively regulates maturation of the autophagosome [34]. 

As is the case in yeast, the mammalian PtdIns 3-kinase is involved in both autophagy and vesicular trafficking toward the lysosome. Discovery of Barkor/Atg14(L) and subsequent research uncovered a similar mechanism that sorts the PtdIns 3-kinase complexes to distinct functions. Barkor/Atg14(L) targets the PtdIns 3-kinase complex to an ER subdomain [35, 36]. The PAS is not identified in mammalian cells. Instead, at least in some cases, autophagosomes are formed inside omegasomes, which are formed at specialized ER subdomains prior to autophagosome formation [37–39]. Barkor/Atg14(L)-mediated ER localization of the PtdIns 3-kinase complex is essential for autophagy [36]. Thus, in both yeast and mammals, Atg14 directs one of the PtdIns 3-kinase complexes to function specifically in autophagy by targeting it to the proposed site of autophagosome formation. The difference in this sorting system between yeast and mammals is the targeting site of the PtdIns 3-kinase complex; yeast Complex I is targeted to the PAS, whereas mammalian autophagy-specific PtdIns 3-kinase complex is targeted to the ER subdomain.

## 5. Structure of Atg14

**Figure 2:**
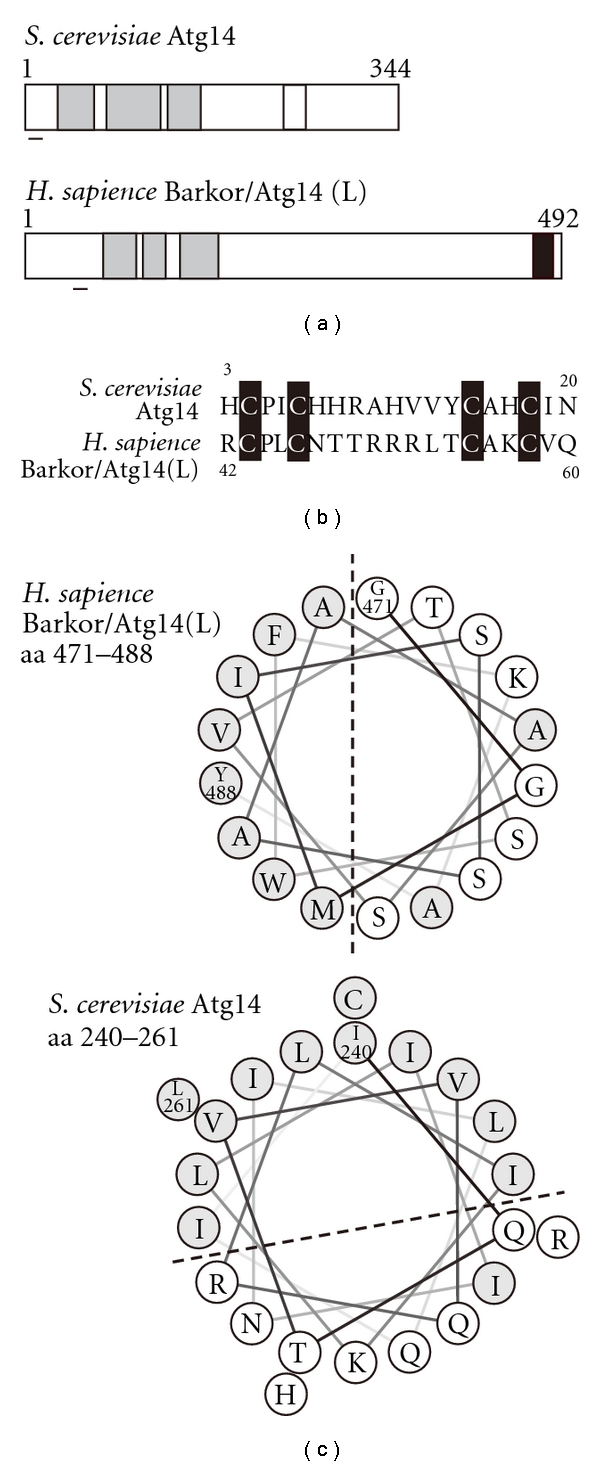
Structure of Atg14. (a) Diagram of yeast Ag14 and human Barkor/Atg14(L). Boxes in gray indicate coiled-coil domains. Bars indicate a position of the conserved cysteine residues. Box in black is the recently found BATS domain containing an amphiphilic helix. Although the BATS domain is not conserved in yeast Atg14, a predicted amphiphilic alpha helix is present within the C-terminal half (white box). (b) The amino acid sequence of the N-terminal motif containing the conserved cysteine residues. The conserved cysteine residues are highlighted. (c) Helical wheel representation of the predicted amphiphilic alpha helix within the C-terminal half of Barkor/Atg14(L) and yeast Atg14. Hydrophobic residues are shown in gray.

The amino acid sequence of Atg14 is not significantly conserved between yeast and mammals, which is consistent with the fact that a sensitive PSI-BLAST method was needed to identify Barkor/Atg14(L). Nevertheless, we can still find similarities between yeast Atg14 and Barkor/Atg14(L) at the levels of local amino acid sequences and of secondary structures. Both yeast Atg14 and Barkor/Atg14(L) contain a cysteine-rich domain at the N-terminal region (Figure 2). Conserved cysteine residues in this region are essential for ER targeting of the PtdIns 3-kinase complex in mammals [36]. At the secondary structural level, both yeast Atg14 and Barkor/Atg14(L) have three predicted coiled-coil domains within the N-terminal half. Deletion analysis of yeast Atg14 revealed that the N-terminal half region containing the coiled-coil domains is sufficient to support autophagic activity, although at a significantly reduced level [32]. The N-terminal half of Atg14 still has the ability to form Complex I and to localize to the PAS. These results suggest that the functions of Atg14—bridging Vps34 and Atg6 and targeting Complex I to the PAS—are exerted by the N-terminal half region. The second coiled-coil domain is involved in interaction with Vps30/Atg6 [32]. Interaction with Vps34 requires the first and the second coiled-coil domains. Atg14 is unstable without the interaction with Vps30/Atg6 and Vps34 through these coiled-coil domains [12]. Although the C-terminal half of Atg14 is not essential for the minimal level of autophagy, it is required to support a normal level of autophagic activity (this issue is discussed later) [32]. Similarly, the coiled-coil domains of Barkor/Atg14(L) play an essential role in autophagy. Coiled-coil domains are required for formation of the autophagy-specific PtdIns 3-kinase complex [18–21]. Of these, the second coiled-coil domain is involved in interaction with Beclin 1 [21, 36], as is the case in yeast Atg14. Endogenous Barkor/Atg14(L) is stabilized by binding to Beclin 1 and Vps34 through the coiled-coil domains [18]. Interestingly, an exogenously expressed Barkor/Atg14(L) variant that lacks the coiled-coils still localizes to the isolation membrane or its precursor, suggesting that other regions are also important for proper localization of Barkor/Atg14(L) [18]. Recently, Fan et al. identified a novel domain, called the Barkor/Atg14(L) autophagosome targeting sequence (BATS) domain, at the C-terminal region of Barkor/Atg14(L) [40]. The BATS domain is essential and sufficient for localizing Barkor/Atg14(L) to the autophagosome. An amphiphilic alpha helix resides at the C-terminus of the BATS domain, and its hydrophobic side plays a crucial role in localizing to the isolation membrane and the autophagosome. It was proposed that the BATS domain senses membrane curvature and binds to the membrane through the hydrophobic side of the amphiphilic alpha helix, thereby, targeting Barkor/Atg14(L) to the isolation membrane to efficiently produce PtdIns(3)*P* there. The BATS domain is conserved in vertebrates but not seen in the yeast Atg14. However, several prediction programs anticipate that a clear amphiphilic helix also resides within the C-terminal half of the yeast Atg14 (Figure 2).

## 6. Involvement of Atg14 in the Regulation of  Autophagic Activity and Autophagosome Size

Mild overexpression of Atg14 increases autophagic activity in yeast [32]. In mammals, overexpression of Barkor/Atg14(L) enhances autophagic activity even under nutrient-rich conditions [36]. Thus, Atg14 seems to be one of the limiting factors regulating autophagic activity. 

**Figure 3:**
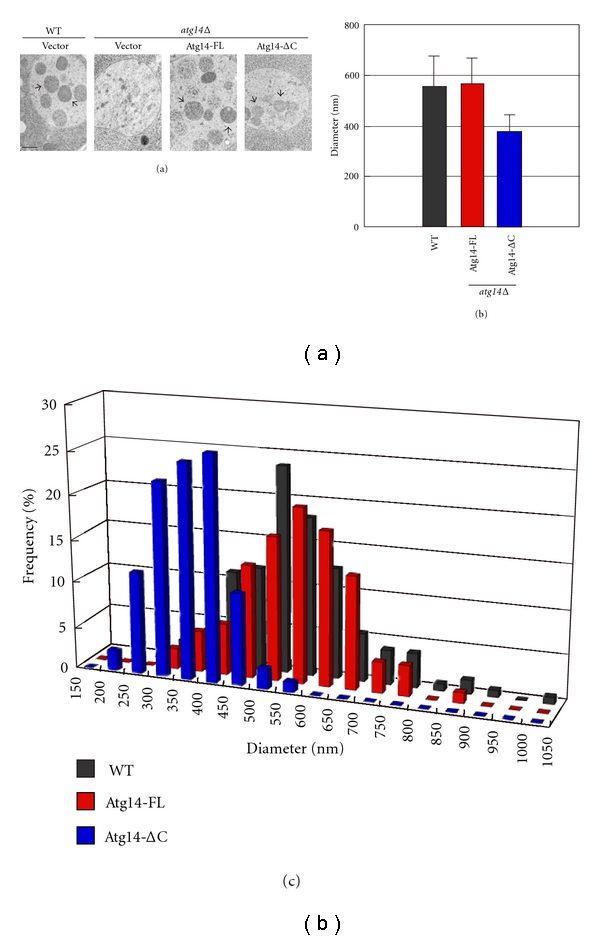
Cells expressing the Atg14-ΔC variant accumulate smaller autophagic bodies. (a) Electron micrographs of autophagic bodies accumulated in BJ2168 cells lacking vacuolar proteases. Cells were cultured for 3.5 h in nitrogen-depleted medium, and the accumulated autophagic bodies (arrow) were observed under an electron microscope. Bar represents 500 nm. (b) Average size of the autophagic body. Autophagic bodies with clear outlines, that is, sliced vertically at the position of the maximum diameter, were chosen, and their diameters were measured. The average autophagic body diameters were 558 nm (*n* = 128), 567 nm (*n* = 86), and 377 nm (*n* = 86) in WT, Atg14-FL, and Atg14-ΔC cells, respectively. Bars indicate standard deviations. (c) Frequency of autophagic bodies with each diameter.

Autophagic activity is reduced in yeast cells expressing an Atg14 variant lacking the C-terminal half (hereafter, Atg14-ΔC) compared to cells expressing the full-length Atg14 (Atg14-FL). Cells expressing the Atg14-ΔC variant accumulate smaller autophagic bodies, indicating that Atg14 has a close relationship with the size of the autophagosome [32]. We performed electron microscopy and measured the diameter of the smaller autophagic bodies accumulated in Atg14-ΔC cells (Figure 3). The average diameter of autophagic bodies accumulated in Atg14-ΔC cells is approximately 66% of that in cells expressing Atg14-FL. Thus, the volume of each autophagic body in Atg14-ΔC cells is estimated to be 29% (the cube of 66%) of that in Atg14-FL cells. Autophagic activity is roughly proportional to the volume of autophagic bodies. Consistent with the estimation based on this electron microscopy, the actual autophagic activity in Atg14-ΔC cells, measured by an established biochemical assay, is approximately 33% of that in Atg14-FL cells [32]. Thus, the C-terminal half of Atg14 is likely to be required to form a normal-sized autophagosome rather than to regulate the number of autophagosomes. How Atg14 regulates the size of autophagosomes is currently unknown. It is possible that the C-terminal half of Atg14 is directly involved in the modulation of autophagosome size. In this sense, it would be interesting to examine whether the amphiphilic helix within the C-terminal half is involved in modulating the curvature of the isolation membrane. Alternatively, the C-terminal half of Atg14 may regulate autophagosome size indirectly through one or more downstream molecules. Deletion of *ATG14* affects the localization of Atg8, the Atg12-Atg5-Atg16 complex, and the Atg2-Atg18 complex [41]. Smaller autophagic bodies are accumulated in cells expressing Atg8 variants with reduced activity [42]. Similarly, the size of the autophagosome correlates with the protein levels of Atg8 [43, 44]. Thus, it is possible that Atg14 regulates autophagosome size indirectly through modulating Atg8 recruitment to the PAS.

As mentioned above, overexpression of Barkor/Atg14(L) activates autophagy in mammalian cells even under nutrient-rich conditions [36], indicating that Barkor/Atg14(L) is one of the key players regulating autophagic activity in mammals. Other subunits of PtdIns 3-kinase complexes are also involved in the regulation of autophagic activity. UVRAG positively regulates maturation of the endosome and the autophagosome. Conversely, Rubicon negatively regulates maturation of the endosome and the autophagosome by sequestering UVRAG from the class C-VPS/HOPS complex [34]. Beclin 1 also plays an important role in regulating autophagy. Beclin 1 interacts with multiple proteins in addition to the core subunits of the PtdIns 3-kinase complexes. One of these, Ambra1, positively regulates autophagy and plays a crucial role in neural development [30]. Beclin 1 also interacts with Bcl-2, an antiapoptotic protein, that is believed to regulate the balance between autophagy and apoptosis [29]. Thus, Beclin 1 may serve as a platform upon which cellular signals converge and function to regulate the crosstalk of multiple processes, including autophagy. This function of regulating the balance of multiple cellular events has not been reported for yeast Vps30/Atg6, which implies that Beclin 1 has obtained this regulatory role during evolution.

In addition to generating PtdIns(3)*P* by the autophagy-specific PtdIns 3-kinase, dephosphorylation of PtdIns(3)*P* also plays an important role in regulating autophagy in mammals. Overexpression of PtdIns(3)*P* phosphatases decreases autophagic activity, while the knockdown or the expression of a dominant-negative form of the phosphatases enhances autophagy [45, 46]. It is currently unclear whether such regulation also occurs in yeast. 

Taken together, Atg14 regulates autophagic activity, at least partially, both in yeast and mammals. However, the Barkor/Atg14(L)-containing PtdIns 3-kinase complex seems to play a more crucial role in determining autophagic activity than yeast Complex I, and the regulation of the Barkor/Atg14(L) complex may have evolved to function in a more sophisticated manner.

## 7. Future Research on Atg14

A conserved function of Atg14 in autophagy is to target the PtdIns 3-kinase complex to the probable site of autophagosome formation. An important problem to be solved is the mechanism whereby Atg14 targets the PtdIns 3-kinase complex to the PAS in yeast and to the ER subdomain in mammals. There are some reports concerning the regulation of PtdIns 3-kinase complex localization. According to a comprehensive analysis of the hierarchy of Atg protein localization, proper targeting of Atg14 is dependent on Atg17 and FIP200 in yeast and mammals, respectively, [35, 41] both of which are scaffold proteins for Atg assembly [47, 48]. The conserved cysteine residues at the N-terminal region of Barkor/Atg14(L) are required for ER localization of Barkor/Atg14(L). Yeast Vps15, a regulatory subunit of PtdIns 3-kinase complexes, can localize to the PAS even in *atg14*Δ cells while Vps34 and Vps30/Atg6 cannot, indicating that Vps15 also contains information related to targeting to the PAS [32]. These fragmented but important results will become the basis for further investigations. 

The function of the N-terminal half of Atg14 has been largely, if not completely, determined. On the other hand, the function of the C-terminal half of Atg14 is still unclear. The C-terminal half of Atg14 is likely involved in forming a normal-sized autophagosome, directly or indirectly. In this sense, it is interesting that the C-terminal BATS domain of Barkor/Atg14(L) binds to the membrane through the hydrophobic surface of the amphiphilic alpha helix. The BATS domain favors highly curved membranes that contain PtdIns(3)*P*, which are considered to be the properties of the isolation membrane. Although the amino acid sequence of the BATS domain is not conserved in yeast Atg14, a clear amphiphilic alpha helix is predicted within the C-terminal half of yeast Atg14. Whether these amphiphilic alpha helixes are involved in the regulation of autophagosome size or not is an interesting issue for future research.
